# Isolation of Proteinase K-Sensitive Prions Using Pronase E and Phosphotungstic Acid

**DOI:** 10.1371/journal.pone.0015679

**Published:** 2010-12-20

**Authors:** Laura D'Castro, Adam Wenborn, Nathalie Gros, Susan Joiner, Sabrina Cronier, John Collinge, Jonathan D. F. Wadsworth

**Affiliations:** MRC Prion Unit and Department of Neurodegenerative Disease, UCL Institute of Neurology, National Hospital for Neurology and Neurosurgery, London, United Kingdom; Federal University of Rio de Janeiro, Brazil

## Abstract

Disease-related prion protein, PrP^Sc^, is classically distinguished from its normal cellular precursor, PrP^C^, by its detergent insolubility and partial resistance to proteolysis. Molecular diagnosis of prion disease typically relies upon detection of protease-resistant fragments of PrP^Sc^ using proteinase K, however it is now apparent that the majority of disease-related PrP and indeed prion infectivity may be destroyed by this treatment. Here we report that digestion of RML prion-infected mouse brain with pronase E, followed by precipitation with sodium phosphotungstic acid, eliminates the large majority of brain proteins, including PrP^C^, while preserving >70% of infectious prion titre. This procedure now allows characterization of proteinase K-sensitive prions and investigation of their clinical relevance in human and animal prion disease without being confounded by contaminating PrP^C^.

## Introduction

Prion diseases are fatal neurodegenerative conditions including kuru, Creutzfeldt-Jakob disease (CJD), Gerstmann-Sträussler-Scheinker syndrome and fatal familial insomnia in humans, scrapie in sheep, bovine spongiform encephalopathy (BSE) in cattle and chronic wasting disease in cervids [1]–[4]. They are characterized by the post-translational conversion of host cellular prion protein (PrP^C^) into an abnormal disease-related isoform designated PrP^Sc^ [1]–[3], [5]. According to the protein-only hypothesis [6], an abnormal PrP isoform is the principal, if not the sole, component of the transmissible prion [1]. It is proposed that PrP^Sc^ is the infectious agent acting to replicate itself with high fidelity by recruiting endogenous PrP^C^ and that the difference between these isoforms lies purely in the monomer conformation and its state of aggregation [1], [2], [5], [7], [8]. The distinct clinical and neuropathological phenotypes that distinguish prion strains are thought to be encoded by pathogenic PrP isoforms with divergent physicochemical properties [9]–[12].

PrP^Sc^ is extracted from affected tissue as highly aggregated material that is not amenable to high-resolution structural techniques. However, Fourier-transform infrared spectroscopic methods and hydrogen/deuterium exchange have shown that PrP^Sc^, in sharp contrast to PrP^C^, has a high β-sheet content [13]–[15]. PrP^Sc^ is covalently indistinguishable from PrP^C^ [1], [7], [16] but can be differentiated from PrP^C^ by its partial resistance to proteolysis and its marked insolubility in detergents [1], [7]. Under conditions in which PrP^C^ exists as a detergent-soluble monomer and is completely degraded by the non-specific protease, proteinase K (PK), PrP^Sc^ exists in an aggregated form with the C-terminal two thirds of the protein showing marked resistance to proteolytic degradation leading to the generation of amino-terminally truncated fragments of di-, mono- and non-glycosylated PrP [1], [7].

Although the molecular diagnosis of prion disease has historically relied upon the detection of PrP^Sc^ using PK, it has recently become apparent that PK-sensitive pathological isoforms of PrP may play an important role in the pathogenesis of prion diseases [17]–[27]. Accordingly, the development of new diagnostic tests that do not rely on PK digestion is essential to fully characterize human and animal prion diseases, and, in this context, the conformation-dependent immunoassay [17], [20] and the amyloid seeding assay [27], [28] both show higher diagnostic sensitivities than can be achieved using PK. Previously, using thermolysin and the mouse Rocky Mountain Laboratory (RML) prion strain, we demonstrated heterogeneity among PK-sensitive disease-related PrP isoforms and found that the majority of prion infectivity (80%) appeared to be associated with a minor fraction of PK-sensitive PrP [25]. Here we now report the development of a biochemical protocol which eliminates PrP^C^ and the majority of brain proteins while preserving both PK-resistant and PK-sensitive prions.

## Results

### Digestion with pronase E degrades PrP^C^ and preserves RML prion titre

As part of ongoing research to identify proteases that offer alternatives to PK, we and others have previously shown that thermolysin can efficiently degrade PrP^C^ while leaving disease-related PrP in full length form [25], [29], [30]. However despite the utility of thermolysin in certain diagnostic applications [25], [29], [30] the degradation of RML prion infectivity by thermolysin obviates its use for isolating PK-sensitive prions [25]. We have therefore continued to examine other proteases for their ability to degrade PrP^C^ while preserving infectious prion titre. Of various proteases recently investigated, pronase E was found to be the most interesting.

**Figure 1:**
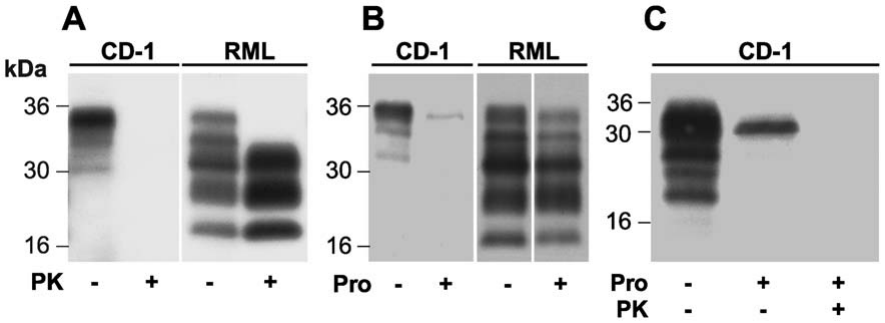
PK and pronase E digestion of mouse brain homogenate. 10% (w/v) brain homogenate from uninfected CD-1 mice (CD-1) and RML prion-infected CD-1 mice (RML) were analysed by immunoblotting in the absence of protease digestion (−) or after digestion (+) with PK (50 µg/ml, 37°C, 1 h) (A) or pronase E (Pro) (100 µg/ml, 37°C, 30 min) (B) or after sequential digestion with pronase E (100 µg/ml, 37°C, 30 min) and PK (50 µg/ml, 37°C, 30 min) (C). Immunoblots were probed with anti-PrP monoclonal antibody ICSM35. The blot shown in panel C has been over-exposed to film to show residual PrP^C^. Apparent molecular masses are indicated in kDa.

Representative digestions of uninfected CD-1 and RML prion-infected mouse brain homogenate with PK or pronase E are shown in Figure 1. Digestion with 50 µg/ml PK at 37°C for 1 h completely degrades PrP^C^ in 10% (w/v) normal CD-1 brain homogenate (Figure 1A) and in 10% (w/v) RML brain homogenate results in a change from a mixture of full-length and truncated PrP species to a characteristic pattern of amino-terminally truncated fragments of di-, mono- and non-glycosylated PrP derived from PrP^Sc^ (Figure 1A). In marked contrast, while digestion with 100 µg/ml pronase E at 37°C for 30 min degraded the majority of PrP^C^ present in uninfected CD-1 brain homogenate (Figure 1B) similar digestion of RML brain homogenate produced no apparent change in the PrP fragment pattern and resulted in only a modest reduction in the overall signal intensity (Figure 1B). In this regard, the activity of pronase E is similar to thermolysin and preserves disease-related PrP in a full length form rather than producing the N-terminally truncated fragments that are generated by PK.

**Figure 2:**
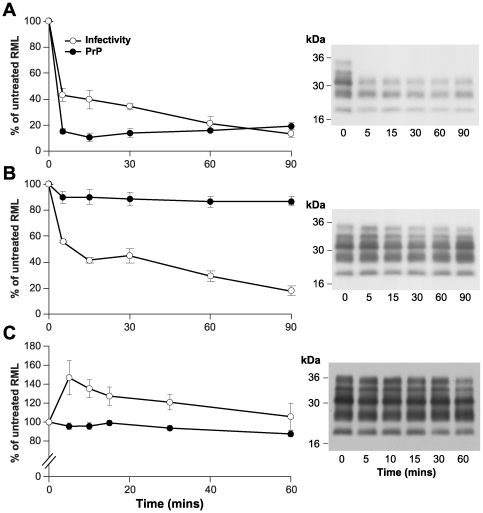
RML prion infectivity and PrP content following digestion with PK, thermolysin or pronase E. 10% (w/v) RML prion-infected brain homogenate was incubated at 37°C with (A) PK (50 µg/ml); (B) thermolysin (100 µg/ml) or (C) pronase E (100 µg/ml) for varying incubation times. For each time point, PrP (filled circles) was measured by ELISA and expressed as a percentage of total PrP present in the untreated sample; mean ± S.E.M. (*n* = 3, PK and thermolysin; *n* = 5, pronase E). RML prion infectivity (open circles) was measured by the Scrapie Cell Assay and expressed as a percentage of total infectivity present in the untreated sample; mean ± S.E.M. (*n* = 3, PK and thermolysin; *n* = 5, pronase E). Immunoblots of the corresponding protease and incubation time point are shown in panels to the right. Blots were probed with anti-PrP monoclonal antibody ICSM35. Apparent molecular masses are shown in kDa. Data in panels A and B have been published previously [25].

Prior to optimizing the parameters of pronase E digestion to completely degrade PrP^C^ we studied what effect pronase E had on RML infectivity using the Scrapie Cell Assay (SCA) [31]. The SCA quantifies RML prion infectivity with an accuracy greater than that of conventional mouse bioassay and provides the ability to measure small percentage changes in infectious prion titre [25], [31]. Recently, we established that digestion of RML prion-infected mouse brain homogenate with PK and thermolysin preserves differing levels of disease-related PrP. Under optimized conditions of proteolysis which degrade PrP^C^, ∼85% or ∼20% of total homogenate PrP resists digestion with thermolysin or PK, respectively [25] (Figure 2A and B). However despite this marked disparity in the concentration of disease-related PrP, ∼80% of prion infectivity was destroyed by either enzyme [25] (Figure 2A and B). These data show that the majority of prion infectivity in RML brain homogenate is sensitive to these proteases and suggest that these prions may be associated with only a minor fraction of total homogenate PrP [25]. Remarkably, in sharp contrast to digestion with either PK or thermolysin, we found that treatment of RML prion-infected brain homogenate with 100 µg/ml pronase E at 37°C did not reduce infectious prion titre even after a 60 min digestion (Figure 2C). Prion infectivity stayed at or above the starting titre throughout the time course, with an increase in infectious titre reproducibly observed after 5–10 min exposure to the protease (Figure 2C). No change in the PrP fragment pattern was observed throughout the digestion (Figure 2C) and the overall reduction in PrP content, to ∼85% of the starting amount, appeared to reflect the degradation of PrP^C^ only. The physical basis for the increase in infectious titre seen after exposure to pronase E remains unexplained, but may reflect changes in PrP aggregate size with concomitant alteration of specific prion infectivity [8] or increased bioavailability of prions following removal of other proteins.

### Enrichment of PK-sensitive prions using pronase E and sodium phosphotungstic acid precipitation

**Figure 3:**
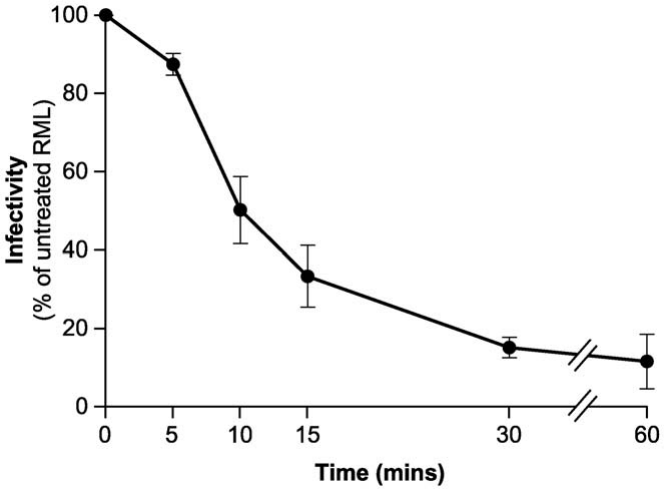
RML prion infectivity following digestion with 1 mg/ml pronase E. 10% (w/v) RML brain homogenate was incubated with pronase E (1 mg/ml at 37°C) for varying incubation times. For each time point RML prion infectivity was measured by the Scrapie Cell Assay and expressed as a percentage of total infectivity present in the untreated sample; mean ± S.E.M. (*n* = 5).

Having established that mild digestion with pronase E does not destroy PK-sensitive prion infectivity we sought to optimize conditions for complete digestion of residual PrP^C^. However this proved to be complicated. Increasing the concentration of pronase E to 1 mg/ml failed to completely eliminate PrP^C^, with ∼10% of the starting concentration remaining after 60 min of digestion time (data not shown). The physical basis for this resistance is difficult to interpret as this minor proportion of PrP^C^ was readily accessible to degradation by subsequent exposure to PK (Figure 1C). Thus this fraction of PrP^C^ is not trapped in an inaccessible form, for example, in a vesicle. Resistance to digestion with pronase E may be attributable to protection through association with other brain proteins which are readily degraded by PK but not by pronase E. Alternatively pronase E may reveal heterogeneity in the aggregation state or conformation of different PrP^C^ isoforms that are present in normal brain as previously documented using other methods [32]–[34]. Regardless of these facts, the effect of higher concentrations of pronase E on RML infectivity was detrimental. Exposure of RML brain homogenate to 1 mg/ml pronase E led to a rapid decrease in prion titre over time and after a 60 min digestion only ∼10% of the starting infectivity remained (Figure 3). Consistent with this loss of infectivity examination of digested material by immunoblotting revealed loss of PrP signal and the transition of PrP fragments to lower molecular mass (data not shown). These results establish that preservation of PK-sensitive prion infectivity is only achieved using a lower concentration (100 µg/ml) of pronase E (Figure 2C).

**Figure 4:**
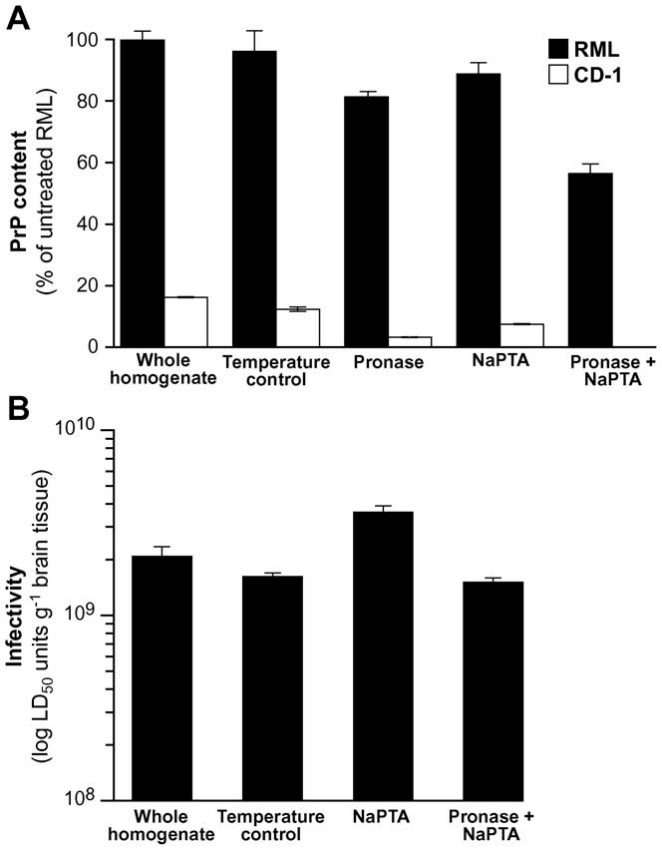
Pronase E-resistant RML prion infectivity is precipitated by NaPTA. 10% (w/v) homogenates from normal CD-1 brain (CD-1, white bars) or RML prion-infected brain (RML, black bars) were either untreated (whole homogenate), incubated at 37°C for 90 min and 20°C for 30 min (temperature control), digested with pronase E at 100 µg/ml, 37°C for 30 min (pronase), precipitated by NaPTA (NaPTA) or sequentially digested by pronase E at 100 µg/ml, 37°C for 30 min and precipitated by NaPTA (pronase + NaPTA). The concentration of PrP in all samples was measured by ELISA (A) and is expressed as a percentage of the total PrP present in the untreated RML samples; mean ± S.E.M. (*n* = 5). RML prion infectivity was measured by the Scrapie Cell Assay (B) and expressed as total infectivity present in the samples; mean ± S.E.M. (*n* = 5).

**Figure 5:**
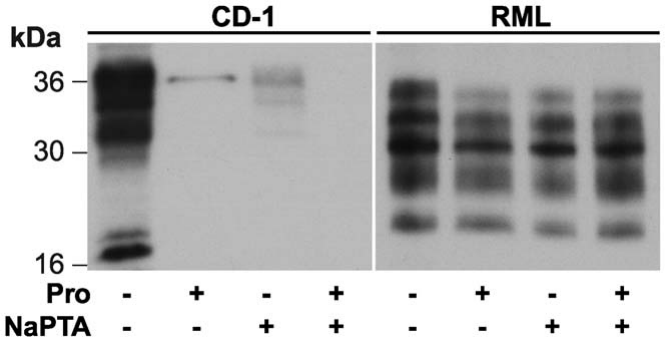
Immunoblot analysis of pronase E digested and NaPTA precipitated mouse brain homogenate. 10% (w/v) homogenates from normal CD-1 brain (CD-1) or RML prion-infected brain (RML) were either untreated (Pro −, NaPTA −), digested by pronase E at 100 µg/ml, 37°C for 30 min (Pro +, NaPTA −), precipitated by NaPTA (Pro −, NaPTA +) or sequentially digested by pronase at 100 µg/ml, 37°C for 30 min and precipitated by NaPTA (Pro +, NaPTA +). Equivalent aliquots of each sample were analysed by immunoblotting using anti-PrP monoclonal antibody ICSM35. Apparent molecular masses are indicated in kDa.

Based on the foregoing results, we sought an alternative method to remove residual PrP^C^ from pronase E-digested brain homogenate. Sodium phosphotungstic acid (NaPTA) is known to selectively precipitate both PK-resistant and PK-sensitive disease-related PrP isoforms from detergent solubilized brain homogenate while leaving the majority of PrP^C^ in the soluble fraction [17], [20], [25]. We therefore applied this protocol to both uninfected CD-1 and RML prion-infected brain homogenate before or after digestion with 100 µg/ml pronase E at 37°C for 30 min and subsequently measured PrP concentration by ELISA or immunoblotting and determined prion infectivity using the SCA. These data established that the residual PrP^C^ that persisted in pronase E-digested normal CD-1 brain homogenate was completely eliminated from the NaPTA precipitated pellet fraction (Figures 4 and 5). Significantly, although the concentration of PrP in the NaPTA pellet fraction from pronase E-digested RML brain homogenate was reduced to ∼60% of the starting level (Figure 4) no significant variation in the fragment pattern of disease-related PrP was observed (Figure 5) and this preparation retained >70% of the starting infectious prion titre (Figure 4) analogous with the levels of infectivity seen in temperature control samples that were simply incubated sequentially at 37°C for 90 min and 20°C for 30 min (Figure 4).

**Figure 6:**
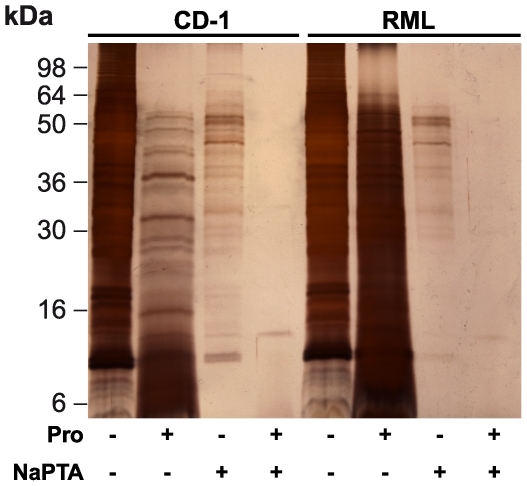
Pronase E digested and NaPTA precipitated mouse brain homogenate evaluated by silver stain SDS-PAGE. 10% (w/v) homogenates from normal CD-1 brain (CD-1) or RML prion-infected brain (RML) were either untreated (Pro −, NaPTA −), digested by pronase E at 100 µg/ml, 37°C for 30 min (Pro +, NaPTA −), precipitated by NaPTA (Pro −, NaPTA +) or sequentially digested by pronase E at 100 µg/ml, 37°C for 30 min and precipitated by NaPTA (Pro +, NaPTA +). The equivalent of 2 µl 10% (w/v) brain homogenate was loaded in each lane and the gel was stained with silver nitrate.

Our findings show that mild digestion with pronase E followed by detergent solubilisation and NaPTA precipitation produces a brain fraction devoid of PrP^C^ that contains >70% of prion infectivity. To assess the relative degree of enrichment of prions achieved by this protocol, we performed SDS-PAGE of treated samples and used silver stain to visualize total protein. This analysis revealed that although pronase E digestion or NaPTA precipitation alone were relatively ineffective in removing brain proteins, when applied in combination a considerable purification of prions (∼150 fold) was achieved with respect to total protein (Figure 6).

## Discussion

Mammalian prions are hypothesized to be self-propagating fibrillar or amyloid forms of PrP in which the ends of the propagating fibrils constitute the infectious entity and that the exponential rise in prion titre during disease progression is a consequence of fiber fragmentation [5], [35], [36]. However, prion diseased brain contains a highly heterogeneous population of abnormal PrP isoforms that may differ with respect to their conformation, aggregate size, potential neurotoxicity and specific prion infectivity [5] and indeed prion strains, rather than constituting a molecular clone, appear to comprise an ensemble or quasispecies maintained under host selection pressure [5], [37]. Due to this heterogeneity, the precise structure of the infectious particle remains unclear. To date, the most highly enriched preparations contain a large molar excess of PrP per infectious unit [8], [38] and it remains unclear what proportion of PrP species are actually infectious [8]. Understanding the ratio of PrP molecules to infectivity is highly convoluted however because of rapid clearance of prions from the brain following intra-cerebral challenge [38] and possible differential clearance kinetics of different size particles. A major confounding issue in this regard is the growing realization that the pathogenesis of both human and animal prion diseases involves the propagation of protease-sensitive disease-related isoforms of PrP [17]–[27] and that a substantial fraction of prion infectivity may be readily sensitive to protease degradation [25]. Indeed, in multiple prion strain/host combinations it now appears that the majority of disease-related PrP may be destroyed by PK under conditions that are typically employed to detect prototypical PrP^Sc^ [20], [23], [25]. PK-sensitive, disease-related, forms of PrP have, to date, been demonstrated by conformation dependent immunoassay [17], [20], [23], [39], by immunoblotting following biochemical purification [18], [40], cold PK digestion [19], immunological capture [21] or limited digestion with thermolysin [25]. However, a simple protocol for isolating PK-sensitive abnormal PrP isoforms in the absence of contaminating PrP^C^ without severely affecting prion titre has not been described. Here we demonstrate that limited digestion of brain homogenate with pronase E followed by detergent solubilisation and selective precipitation with NaPTA yields a preparation that retains the majority of the infectious prion titre but is devoid of PrP^C^ and the majority of other brain proteins. It is envisaged that this protocol may have widespread utility in improving diagnostic sensitivity from analysis of brain and other tissues in human and animal prion diseases and may now also simplify isolation and biophysical characterization of PrP structures having the highest specific prion infectivity. Methods for isolating slowly sedimenting, low density PrP^Sc^ aggregates of high specific prion infectivity from different prion strains have recently been described [41] however the authors reported that an abundance of PrP^C^ and other components in these slowly sedimenting fractions prevented detailed characterization of the most infectious PrP particles. Similar comparative fractionation of pronase E and NaPTA precipitated samples before or after PK digestion would now be expected to inform upon the density distribution of both PK-sensitive and PK-resistant abnormal PrP species and enable measurement of specific prion infectivity without being confounded by contaminating PrP^C^. Further biophysical characterization of slowly sedimenting PK-sensitive abnormal PrP species may reveal whether these are predominantly composed of small PrP oligomers or larger PrP aggregates associated with lipid.

## Materials and Methods

### Ethics Statement

Work with animals was performed under licence granted by the UK Home Office (Project Licence number 70/6454) and conformed to University College London institutional guidelines.

### Prion-infected tissues

All procedures were carried out in a microbiological containment level 3 facility with strict adherence to safety protocols. Brains from 400 terminally-affected CD-1 mice infected with the RML prion strain [42] were homogenized in Dulbecco's phosphate buffered saline lacking Ca^2+^ or Mg^2+^ ions (D-PBS) using tissue grinders to produce two pools of 10% (w/v) RML brain homogenate (designated I6200 and I8700). Both I6200 and I8700 showed comparable prion infectivity titres in the Scrapie Cell Assay (SCA) [25], [31] and the titre of I6200 measured by bioassay in CD-1 mice was 10^9.3^ intracerebral LD_50_ units/g of brain [25]. Three batches of 18 brains from uninfected CD-1 mice were homogenized to produce three pools of ∼90 ml of 10% (w/v) normal CD-1 brain homogenate (designated I7219, I8402 or I10340). Pooled homogenates were dispensed as aliquots and stored at −70°C until use.

### Enzymatic digestion

Protease type XIV (synonyms: pronase E, actinase E) from *Streptomyces griseus* was obtained as a lyophilized powder from Sigma-Aldrich (Prod. No. P5147). The specific enzymatic activity is approx 4 units/mg (where 1 unit liberates 1.0 µmol of tyrosine/min at pH 7.5 at 37°C using casein as a substrate). Proteinase K (EC 3.4.21.64) from *Tritirachium album limber* was obtained freeze-dried from Merck. The specific enzymatic activity is approx 30 Anson units/g (where 1 Anson unit is the amount of enzyme that liberates 1 mmol of Folin-positive amino acids/min at pH 7.5 and 35°C, using haemoglobin as a substrate). Thermolysin (EC 3.4.24.27) from *Bacillus thermoproteolyticus rokko* was obtained freeze-dried from Sigma–Aldrich. The specific enzymatic activity is 50–100 units/mg of protein (where 1 unit liberates 1 µmol of tyrosine/min at pH 7.5 and 37°C using casein as a substrate). Stock solutions of 1 mg/ml thermolysin and PK and 1 mg/ml and 10 mg/ml pronase E were prepared in water and aliquots were stored at −70°C. Aliquots of 10% (w/v) brain homogenates in D-PBS were digested for variable time periods at 37°C with thermolysin (at a final protease concentration of 100 µg/ml), with PK (at a final protease concentration of 50 µg/ml) or with pronase E (at final protease concentrations varying between 100 µg/ml to 1 mg/ml). Aliquots of the digested brain homogenates or untreated control samples were snap-frozen in liquid nitrogen for infectivity studies, or processed immediately for analysis by either immunoblotting or ELISA.

### PrP immunoblotting

Aliquots of brain homogenate were mixed with an equal volume of 2 x SDS sample buffer (125 mM Tris/HCl (pH 6.8), 20% (v/v) glycerol, 4% (w/v) SDS, 4% (v/v) 2-mercaptoethanol and 0.02% (w/v) bromophenol blue containing 8 mM 4-(2-aminoethyl) benzenesulphonyl fluoride) and immediately transferred to a 100°C heating block for 10 min. Samples were analysed by electrophoresis in 16% (w/v) Tris-glycine gels and immunoblotting as described previously [43]. Blots were blocked in PBS containing 0.05% (v/v) Tween 20 (PBST) and 5% (w/v) non-fat dried skimmed milk powder and probed with the anti-PrP monoclonal antibody ICSM 35 (D-Gen Ltd, London) at 0.2 µg/ml final antibody concentration in PBST. Blots were developed using alkaline-phosphatase-conjugated anti-mouse IgG secondary antibody and chemiluminescent substrate CDP-Star (Tropix), and visualized on BiomaxMR film (Kodak) as described previously [43].

### Silver staining

SDS-PAGE gels were fixed in 40% (v/v) ethanol and 10% (v/v) glacial acetic acid before silver staining using the PlusOne silver stain kit (GE Healthcare, Little Chalfont, UK) according to the manufacturers instructions. Gels were photographed on a light box using a camera stand (Kaiser RS-1) and a Nikon D80 with 18–135 mm zoom lens or a Nikon Coolpix P6000.

### ELISA

Methods were performed as described previously [44] with adaptations. Brain homogenates were treated at 37°C with thermolysin (100 µg/ml final protease concentration), PK (50 µg/ml final protease concentration) or pronase E (100 µg/ml or 1 mg/ml final protease concentrations) for a range of incubation times. Subsequently, 10 µl aliquots of these samples or untreated brain homogenate and temperature controls were adjusted with 10 µl of 4% (w/v) SDS and heated at 100°C for 10 min. Samples were centrifuged at 100×*g* for 30 seconds before addition of 600 µl of 50 mM Tris/HCl (pH 8.4) containing 2% (v/v) Triton X-100, 2% (w/v) sodium lauroylsarcosine and 2% (w/v) bovine serum albumin (Fraction V, protease free, Sigma–Aldrich). Aliquots (50 µl) were transferred into the wells of microtitre plates (Microlon 96W, Greiner Bio-One) containing immobilized anti-PrP monoclonal antibody ICSM18 (250 ng/well; D-Gen Ltd, London). After incubation at 37°C for 1 hr with constant agitation, wells were washed with 3×300 µl of PBST using an automated microplate washer, followed by the addition of 100 µl of PBS containing 1% (v/v) Tween 20 and 1 µg/ml biotinylated anti-PrP monoclonal antibody ICSM 35. Following incubation at 37°C for 1 h with constant agitation, wells were washed as detailed above, followed by the addition of 100 µl of PBS containing 1% (v/v) Tween 20 and a dilution of streptavidin–horseradish-peroxidase conjugate (1∶10000 dilution; Dako). After incubation at 37°C for 30 min with constant agitation, wells were washed with 4×300 µl of PBST. Wells were developed with 100 µl of QuantaBlu working solution (Pierce) and the reactions were stopped by the addition of 100 µl of QuantaBlu stop solution (Pierce). Fluorescence (*λ*ex  = 340 nm, *λ*em  = 420 nm) was measured on a Tecan spectra image microplate reader.

### Sodium phosphotungstic acid (NaPTA) precipitation

The use of NaPTA was adapted from Safar and colleagues [17] as described previously [43]. Briefly, 50 µl aliquots of 10% (w/v) brain homogenate were incubated at 37°C for 30 min in the absence or presence of pronase E (100 µg/ml final concentration). Pronase E digestion was stopped by the addition of 1 µl 0.5 M EDTA pH 8.0 prepared in water. Samples were incubated with 50 µl D-PBS containing 4% (w/v) sodium lauroylsarcosine (sarkosyl) at 37°C for 30 min with constant agitation. Samples were subsequently adjusted with 8.5 µl of a stock solution of 4% (w/v) NaPTA prepared in water and adjusted to pH 7.4 (magnesium chloride was omitted) to give a final concentration in the sample of 0.3% (w/v). Samples were incubated at 37°C for 30 min with constant agitation before centrifugation at 16,100×*g* for 30 min in a microfuge. After careful isolation of the supernatant, the pellets were resuspended to 50 µl with D-PBS containing 0.1% (w/v) sarkosyl. Aliquots of the resuspended pellets were snap-frozen in liquid nitrogen for infectivity studies, or processed immediately for analysis by either immunoblotting or ELISA.

### Scrapie cell assay

High-sensitivity cell-culture assays for RML prion infectivity were performed as described previously [25], [31]. Briefly, PK1 cells, an N2a sub-clone highly susceptible to infection with RML prions, were exposed for 3 days in 96-well plates to serial dilutions (10^−4^ and 10^−5^) of 10% (w/v) RML brain homogenate before or after protease digestion or precipitation with NaPTA. Serial dilution of untreated 10% (w/v) RML brain homogenate (3×10^−4^ to 1×10^−6^) of known infectivity titre was performed in parallel. The prion infectivity titre in experimental samples was deduced from the reference preparation [31].

### Statistical analysis

All experiments were conducted at least three times. Figures show representative data and means ± S.E.M.
